# Polymer-Infiltrated Ceramic Network Versus Smart Bioactive Self-Curing Composite for Cervical Restorations in Professional Ballet Dancers: A 24-Month Split-Mouth Randomized Controlled Trial

**DOI:** 10.3390/medicina62061141

**Published:** 2026-06-11

**Authors:** Maria Timoshina, Sergey Mironov, Alexey Dorofeev, Alla Shakaryants, Svetlana Danshina, Ksenia Zakharova, Ksenia Grishaeva, Aglaya Kazumova, Anton Timoshin, Andrey Sevbitov

**Affiliations:** Borovskiy Institute of Dentistry, Sechenov First Moscow State Medical University (Sechenov University), 8/2 Trubetskaya St., Moscow 119991, Russia; timoshina_m_d@staff.sechenov.ru (M.T.); mironov_s_n_1@staff.sechenov.ru (S.M.); dorofeev_a_e@staff.sechenov.ru (A.D.); davidyants_a_a@staff.sechenov.ru (A.S.); danshina_s_d@staff.sechenov.ru (S.D.); zakharova_k_e@staff.sechenov.ru (K.Z.); grishaeva_k_a@staff.sechenov.ru (K.G.); timoshin_a_v@staff.sechenov.ru (A.T.); sevbitov_a_v@staff.sechenov.ru (A.S.)

**Keywords:** polymer-infiltrated ceramic network, PICN, self-curing composite, cervical restoration, abfraction defect, ballet dancers, randomised controlled trial, split-mouth design, marginal integrity, USPHS criteria

## Abstract

*Background and Objectives*: Professional ballet dancers endure high occlusal loads, increasing cervical defect prevalence. Conventional composites fail frequently under such conditions. This randomized clinical trial (RCT) compared 24-month performance of a polymer-infiltrated ceramic network (PICN, VITA Enamic) versus a self-curing bioactive composite (Stela) for cervical restorations. *Materials and Methods*: Twenty professional ballet dancers (40 cervical defects: 21 carious, 19 abfraction) were enrolled in a paired split-mouth RCT. Each received one PICN inlay and one self-curing composite restoration on two non-adjacent defects. Restorations were assessed at 6, 12, and 24 months using United States Public Health Service (USPHS) criteria (primary: marginal integrity) and a dye penetration test. Secondary outcomes included secondary caries, hypersensitivity, and Oral Health Impact Profile-14 (OHIP-14). Statistical tests: McNemar, Fisher’s exact, Kaplan–Meier, log-rank (α = 0.05). *Results*: At 24 months, marginal integrity (USPHS Alpha) was maintained in 91% of PICN restorations for carious defects and 89% for abfraction defects, compared to 70% and 50% for self-curing composite, respectively. No PICN restoration failed (0%). Self-curing composite failures were 20% (carious) and 30% (abfraction) (exploratory uncorrected *p* = 0.031; non-significant after correction). Dye penetration was lower for PICN in abfraction defects (11% vs. 60%, adjusted *p* = 0.048) but not in carious defects (9% vs. 30%, adjusted *p* = 0.317). Kaplan–Meier survival favoured PICN (log-rank *p* = 0.001); 24-month survival probability: PICN 100% (95% CI: 83–100%), self-curing composite 75% (95% CI: 55–95%). No secondary caries or serious adverse events occurred. *Conclusions*: PICN hybrid ceramic provided superior marginal integrity and zero failures over 24 months in cervical restorations of professional ballet dancers, outperforming the self curing composite. Within this high-risk population, PICN inlays are recommended for abfraction defects. However, because the study was conducted exclusively in professional ballet dancers, direct extrapolation to the general population should be made with caution. The self-curing composite may be considered for carious defects when light curing is problematic, but patients should be informed of higher failure risk. Longer studies are needed.

## 1. Introduction

Professional ballet dancers are exposed to extreme and sustained physical demands from early childhood [1]. Constant tension of facial and masticatory muscles, combined with parafunctional clenching, leads to occlusal disturbances, accelerated tooth wear, and a high prevalence of temporomandibular disorders [2,3]. These loading patterns generate bending stress in the cervical region, promoting loss of calcium ions from hydroxyapatite and contributing to abfraction lesions [4].

Caries prevalence among ballet dancers approaches 100% across all age groups, with the DMFT index rising progressively with age and professional experience [5]. Poor oral hygiene, low motivation for preventive care, and a high-carbohydrate diet further increase susceptibility to both carious (Class V) and non-carious (abfraction) cervical defects [6,7,8].

Conventional treatment relies on direct nano-hybrid composite restorations. However, under high cyclic occlusal loads, these show high rates of marginal deterioration, secondary caries, and premature failure [4]. The mismatch in elastic modulus between conventional composites and tooth structure leads to stress concentration at the tooth–restoration interface, compromising long-term marginal integrity.

Recent advances have introduced two innovative material classes that may overcome these limitations.

Polymer-infiltrated ceramic network (PICN) materials (hybrid/resin-matrix ceramics) combine a favourable elastic modulus and damage tolerance with wear resistance, mimicking the biomechanical properties of natural dentin and enamel [9]. Long-term prospective studies and systematic reviews have confirmed excellent survival rates and marginal stability of PICN restorations in high-stress applications, including severe tooth wear and cervical defects [10,11,12,13,14,15,16,17,18].

Smart bioactive self-curing composites use a hydroperoxide-based initiator system that, according to in vitro studies, virtually eliminates polymerisation stress and achieves a gap-free interface [19,20]. Stela (SDI, Australia) additionally contains ion-releasing glass fillers (ionglass™) that release calcium, fluoride, and strontium ions, potentially promoting remineralisation and providing anti-cariogenic benefits [19,21,22]. Clinical studies have shown acceptable performance of Stela in posterior restorations [23,24,25,26,27,28,29].

Despite the promise of both material classes, no clinical study has directly compared a PICN hybrid ceramic with a smart bioactive self-curing composite for cervical restorations. Moreover, no RCT has evaluated these materials specifically in professional ballet dancers—a population exposed to predictable extreme masticatory loads, providing a challenging test scenario for restorative durability [3,5]. It is important to recognise that the two materials differ not only intrinsically but also in clinical workflow (indirect versus direct technique). Therefore, this trial compares two complete restorative protocols rather than materials in isolation.

Aim of the study: To compare the clinical performance of a PICN hybrid ceramic (VITA Enamic) versus a smart bioactive self-curing composite (Stela) for cervical restorations in professional ballet dancers over 24 months in a split-mouth RCT.

Hypothesis: PICN restorations will demonstrate superior marginal integrity and lower failure rates compared with the self-curing composite; however, the self-curing composite may offer advantages in simplified placement and reduced polymerisation stress.

## 2. Materials and Methods

### 2.1. Study Design

This study was designed as a prospective, split-mouth, randomized controlled trial with a 24-month follow-up. The protocol was approved by the Local Ethics Committee of I.M. Sechenov First Moscow State Medical University (Protocol No. 22/21, 9 December 2021) and was registered at ClinicalTrials.gov (NCT07598305). The trial was conducted in accordance with the Consolidated Standards for Reporting Trials (CONSORT) 2010 guidelines [30] for randomized trials and followed the Oral Health Statistics (OHStat) recommendations [31] for reporting of dental research. All participants provided written informed consent before enrolment.

### 2.2. Participants and Eligibility Criteria

Participants were recruited from the Centre for Sports and Ballet Trauma and Rehabilitation of the Priorov National Medical Research Centre of Traumatology and Orthopaedics (Moscow, Russian Federation). Eligible individuals were current or retired professional ballet dancers with at least ten years of professional experience, aged 18 to 50 years, and presenting at least two non-adjacent cervical defects (Class V carious lesions or abfraction defects) located in different quadrants. Cervical defects were classified as carious (Class V) or non-carious (abfraction) based on clinical and radiographic criteria (presence of caries detected visually and radiographically, absence of caries in abfraction lesions). DMFT index was assessed according to World Health Organization criteria: a tooth was considered decayed (D) if caries was detected visually or radiographically, missing (M) if extracted due to caries, and filled (F) if a permanent restoration was present with no evidence of recurrent caries. Each defect had a depth of at least 1.5 mm and a width not exceeding 4 mm. Teeth had to be vital, free of active periodontal disease (probing depth ≤ 3 mm), and without clinical or radiographic signs of pulpal pathology.

Exclusion criteria were severe bruxism requiring occlusal splint therapy (mild to moderate bruxism or parafunctional clenching without splint indication was allowed, as these are typical for the target population), uncontrolled systemic diseases (e.g., diabetes mellitus, autoimmune disorders), pregnancy or lactation, known allergic reactions to any component of the restorative materials, and inability to attend scheduled follow-up visits.

After initial assessment, 20 of 26 screened individuals met the eligibility criteria and were enrolled.

### 2.3. Randomization, Allocation Concealment, and Blinding

For each participant, the two eligible cervical defects were randomly allocated to one of the two restorative materials using a computer-generated randomization sequence with a 1:1 allocation ratio and a block size of four. The sequence was generated by an independent statistician using randomizer.org. Stratification by defect type was not feasible because the two defects within a participant could differ in type, and the number of possible combinations was insufficient for blocked stratification. The balance of defect types between material groups was assessed post hoc (see Table 1). Allocation concealment was achieved by sequentially numbered, opaque, sealed envelopes that were opened immediately before the restorative procedure. The envelopes were prepared by a researcher not involved in patient treatment or outcome assessment.

Blinding was applied as follows: the outcome assessors—two calibrated dentists who did not perform any of the restorations—were kept unaware of the material assignment. Both USPHS and dye penetration assessments were performed by these blinded examiners. Participants were not informed which material was placed on which tooth. Although the two restorative workflows differed (indirect PICN requiring two visits versus direct self-curing composite placed in one visit), each participant underwent separate appointments for each defect, and the order of treatments was randomized. Consequently, participants could not reliably attribute a specific procedural length or number of appointments to a particular material, preserving blinding. Patient-reported outcomes (OHIP-14) were self-administered by participants independently, without involvement of the operating dentist or outcome assessors. The operating dentist who performed all restorations could not be blinded owing to the inherently different handling characteristics of the two materials.

### 2.4. Sample Size Calculation

The required sample size was calculated a priori using G*Power software (version 3.1.9.7; Heinrich Heine University, Düsseldorf, Germany). Based on pilot data, a difference of 30% in the proportion of restorations demonstrating marginal defects was anticipated between the two groups (expected 40% in the control group versus 10% in the experimental group). Using a two-sided paired proportions test (split mouth design) with α = 0.05 and β = 0.20 (power = 80%), the required number of paired defects was 28. To account for an anticipated 20% dropout rate at 24 months, 40 defects (20 per material group) from 20 participants were included in the final analysis. The primary analysis was powered to detect an overall difference between materials without stratification by defect type. Comparisons within subgroups (carious vs. abfraction defects) were not accounted for in the sample size calculation and are therefore exploratory. Results from subgroup analyses should be interpreted with caution due to limited statistical power.

### 2.5. Restorative Procedures

All restorative procedures were performed by a single experienced operator under local anaesthesia (4% articaine with epinephrine 1:100,000) (A.V.S., 12 years of clinical experience in restorative dentistry). Before the study, the operator was calibrated on 10 additional cervical defects (not included in the trial). For each cervical defect, cavity preparation was kept minimally invasive: carious lesions underwent conventional caries removal with a round bur, application of a caries detector, and acid etching (37% orthophosphoric acid, 15 s for enamel, 10 s for dentin). No additional mechanical preparation was performed for abfraction defects; the existing lesion shape was used as the cavity form for the indirect restoration. This conservative approach is justified because the natural wedge shape of abfraction lesions provides adequate retentive geometry for adhesive bonding, while additional bur preparation would unnecessarily sacrifice tooth structure. Impression-taking is feasible due to the absence of sharp undercuts, and the smooth lesion surface allows for predictable seating of the indirect restoration.

Group A—PICN hybrid ceramic (VITA Enamic, VITA Zahnfabrik, Bad Säckingen, Germany). After cavity preparation, an impression was taken using polyvinyl siloxane, and the inlay was fabricated by a commercial dental laboratory using a CEREC CAD/CAM system (software version 5.1, Dentsply Sirona, Charlotte, NC, USA) with VITA Enamic blocks; the restoration design was reviewed and approved by the operator before milling. At the second visit, the inlay was bonded as follows. The intaglio surface of the PICN inlay was etched with 5% hydrofluoric acid (VITA Ceramics Etch, VITA Zahnfabrik) for 60 s, thoroughly rinsed with water for 30 s, air-dried, and then silanated (Monobond Plus, Ivoclar Vivadent, Schaan, Liechtenstein) for 60 s. The cavity was etched with 37% orthophosphoric acid for 15 s on enamel and 10 s on dentin, rinsed, and gently air-dried leaving the dentin slightly moist. A dual-cure, self-adhesive resin cement (G-CEM ONE, GC Corporation, Tokyo, Japan) was applied to the intaglio surface of the inlay. The inlay was seated under finger pressure, excess cement was removed with a brush, and each margin was tack-cured for 2 s. After removing remaining excess cement, final light curing was performed for 20 s per surface (wavelength 450–470 nm, intensity ≥ 1000 mW/cm^2^). The cement self-cures additionally in the absence of light, ensuring complete polymerization even in deep cervical margins.

Group B—Smart bioactive self-curing composite (Stela, SDI, Bayswater, VIC, Australia). Stela is a self-curing bulk-fill composite that utilizes a hydroperoxide-based initiator system. The manufacturer’s two-step protocol was applied: Stela Primer was applied to the cavity for 15 s, followed immediately by bulk placement of Stela (single increment). No light-curing was required. After setting, finishing was performed with fine diamond burs and polishing discs.

After placement of each restoration, occlusal contacts were checked with articulating paper (12 μm thickness) in centric occlusion and during lateral excursions. In cases where a premature or interceptive contact was detected on the restored tooth, it was carefully adjusted using a fine diamond bur and repolished with silicon polishing points. This step was performed to eliminate occlusal interferences that could otherwise concentrate bending stresses on the cervical margin, particularly relevant in patients with high parafunctional loads. Final polishing was then completed with silicon polishing points.

### 2.6. Outcome Assessment

Patients were recalled for clinical evaluation at 6, 12, and 24 months after restoration placement. Two calibrated examiners (intra-examiner kappa = 0.86; inter-examiner kappa = 0.82), who were blinded to the material assignment, performed the assessments independently. The same two examiners were also calibrated for the dye penetration test in a separate session using 20 additional cervical restorations (not included in the trial), achieving intra-examiner kappa = 0.84 and inter-examiner kappa = 0.79.

Primary outcomes (co-primary) included:

Marginal integrity—evaluated using the modified United States Public Health Service (USPHS) criteria (Alpha, Bravo, Charlie, Delta) with a dental mirror, probe, and additional light source.

Dye penetration—assessed by the Borovsky–Aksamit test: a 2% methylene blue solution was applied to the restoration margin for 2 min, then rinsed for 10 s; the presence (+) or absence (–) of dye penetration at the tooth–restoration interface was recorded. Scoring was performed under ×2.5 magnification loupes. While not a conventional primary endpoint in clinical trials, the Borovsky–Aksamit test provides a simple, reproducible, and inexpensive method to detect subclinical marginal gaps that may precede overt restoration failure. It has been validated in multiple clinical studies and correlates with early marginal deterioration.

Secondary outcomes included the development of secondary caries (clinical and radiographic assessment), postoperative hypersensitivity (measured on a visual analogue scale), and patient satisfaction (using the Oral Health Impact Profile-14 (OHIP-14) questionnaire).

### 2.7. Statistical Analysis

Statistical analysis was performed using StatTech v. 3.1.6 (Stattech LLC, Moscow, Russia). The Shapiro–Wilk test was used to assess normality of continuous data. For paired binary outcomes (marginal integrity defects and dye penetration) at each time point, McNemar’s test was applied. For inter-group comparisons of failure rates, Fisher’s exact test was used when expected cell counts were below 5; otherwise, the chi-square test was employed. Differences in failure rates between carious and abfraction defects within each material group were assessed using the chi-square test or Fisher’s exact test, as appropriate. Ninety-five percent confidence intervals (CI) for differences in proportions were calculated using the Wilson method. To control type I error, we applied the Holm–Bonferroni correction sequentially: first across the three follow-up time points (6, 12, and 24 months) for each outcome separately (adjusted α = 0.017 per comparison), and second across the two co-primary outcomes (marginal integrity and dye penetration), with an adjusted significance level of α = 0.025 for each co-primary outcome. No further correction was applied for comparisons between defect types (carious vs. abfraction) or for the descriptive subgroup analyses, as these were designated as exploratory. The uncorrected *p*-values are reported for exploratory comparisons, and readers should interpret them with caution. All *p*-values reported for primary outcomes in Table 2 and Table 3 are corrected for two comparisons unless stated otherwise. Restoration survival (failure defined as USPHS Charlie or Delta) was analysed using Kaplan–Meier survival curves, and the log-rank test was used to compare survival distributions between the two groups. A two-tailed *p*-value < 0.05 was considered statistically significant. To account for within-patient correlation in survival analysis, a sensitivity analysis using a shared-frailty Cox model confirmed the findings. All analyses followed the intention-to-treat principle. No missing data occurred; intention-to-treat (ITT) analysis was equivalent to per-protocol analysis. All statistical tests were two-tailed, and a *p*-value < 0.05 was considered statistically significant for primary analyses after applying the corrections described above.

## 3. Results

### 3.1. Participant Flow and Baseline Characteristics

Of the 26 professional ballet dancers initially assessed for eligibility, 20 met the inclusion criteria and were enrolled. All 20 participants (mean age 34.2 ± 8.7 years; 14 females, 6 males) completed the 24-month follow-up without any dropouts. No adverse events or postoperative hypersensitivity requiring intervention were reported in either group.

A total of 40 cervical defects (21 carious lesions, 19 abfraction defects) were restored (20 with PICN hybrid ceramic, 20 with self-curing composite). Within each material group, the distribution of defect types was balanced (PICN: 11 carious, 9 abfraction; self-curing: 10 carious, 10 abfraction). Regarding lesion type combinations within participants: 12 participants had two carious defects (both Class V), 8 participants had two abfraction defects, and no participant had mixed defect types (one carious and one abfraction). Thus, each participant contributed two defects of the same type, which further supports the validity of paired analyses within defect type. Baseline characteristics are reported at the patient level because of the split-mouth design. All 20 patients received both materials (one on each of two non-adjacent cervical defects). The distribution of defect types between the two material groups was balanced (Table 1).

Among the 20 participants, 14 were current professional ballet dancers (still actively performing and training) and 6 were retired dancers (mean time since retirement 4.3 ± 2.1 years).

**Figure 1:**
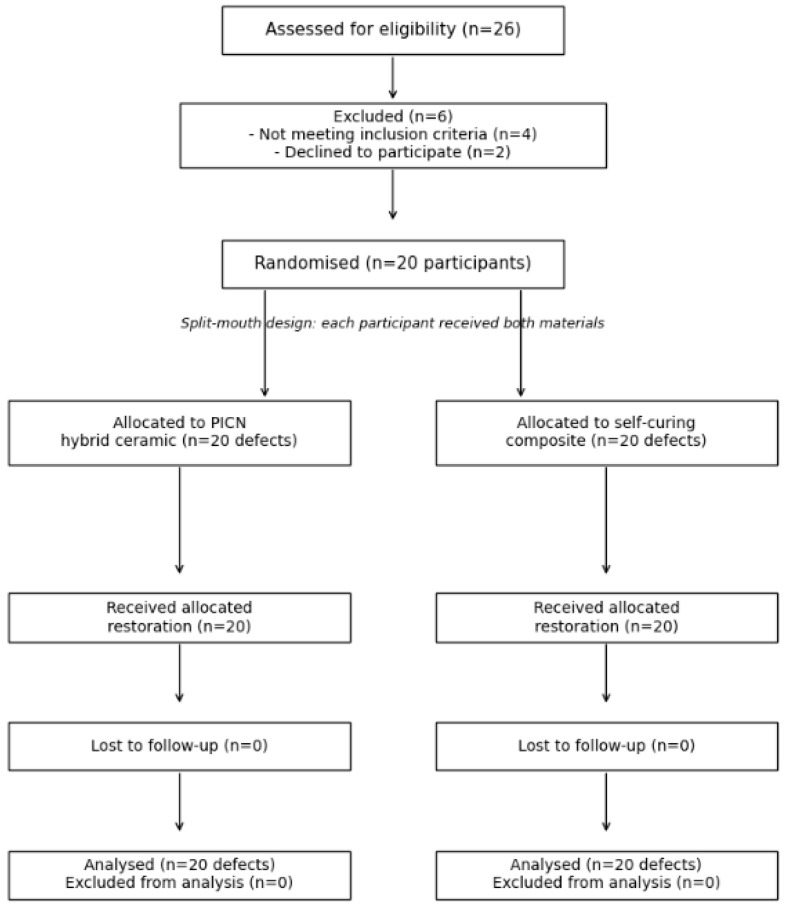
CONSORT 2010 flow diagram of participant enrolment, randomisation, follow-up, and analysis.

Figure 1 presents the CONSORT 2010 flow diagram for participant enrolment, allocation, follow-up, and analysis. Of 26 professional ballet dancers assessed, 6 were excluded, and 20 were randomised. All 20 participants completed the 24-month follow-up with no dropouts.

### 3.2. Marginal Integrity (USPHS Criteria) by Defect Type

Marginal integrity was assessed at 6, 12, and 24 months using USPHS criteria (Alpha = excellent, Bravo = visible gap not reaching dentino-enamel junction (DEJ), Charlie = gap to dentine, Delta = restoration loss). Failure was defined as Charlie or Delta. Marginal deterioration in this manuscript refers to USPHS Bravo or worse (visible gap or greater), whereas failure is strictly defined as Charlie or Delta.

At 24 months, no PICN restoration failed in either carious or abfraction defects. In contrast, self-curing composite restorations showed a cumulative failure rate of 20% for carious defects and 30% for abfraction defects. The absolute difference in failure rates between defect types within the self-curing group was 10% (30% minus 20%; 95% CI for the difference: −5% to 45%; uncorrected *p* = 0.031; after correction *p* = 0.093, not significant, chi square test). Importantly, this comparison was not pre-specified in the primary analysis plan and is exploratory; the *p*-value is not adjusted for multiple subgroup testing. Table 2 presents the detailed distribution.

Accordingly, all comparisons between defect types within material groups are presented as exploratory analyses.

**Figure 2:**
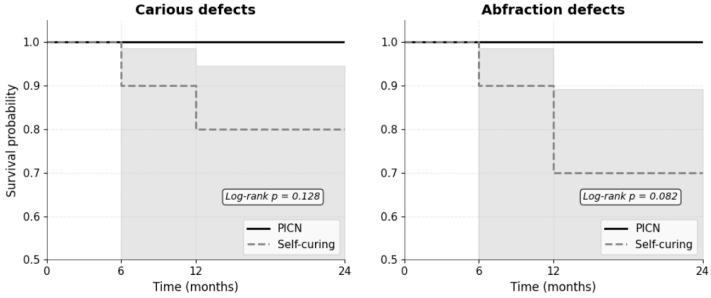
Kaplan–Meier survival analysis. Shaded areas represent 95% confidential intervals (CI). PICN = polymer-infiltrated ceramic network.

Kaplan–Meier survival analysis (Figure 2) confirmed significantly better survival for PICN restorations overall (log-rank *p* = 0.001), with separation of curves evident from 6 months.

No censoring occurred due to 0% dropout. In the self-curing composite group, two failures occurred at 12 months (one carious, one abfraction) and three additional failures at 24 months (one carious, two abfraction). Numbers at risk were: PICN—20 at 0, 6, 12, and 24 months; self-curing composite—20 at 0 months, 20 at 6 months, 18 at 12 months, and 15 at 24 months. The hazard ratio for failure of self-curing composite compared to PICN was 15.2 (95% CI: 1.9 to infinity; *p* = 0.002) using a Cox proportional hazards model stratified by patient.

### 3.3. Dye Penetration Test (Borovsky–Aksamit)

Dye penetration at the tooth-restoration interface increased over time. At 24 months, it was significantly lower for PICN restorations only in abfraction defects (adjusted *p* = 0.048), while the difference in carious defects was not significant (adjusted *p* = 0.317). As shown in Table 3, dye penetration was higher for self-curing composite than for PICN in both defect types, but the difference was statistically significant only for abfraction defects (adjusted *p* = 0.048). Results are summarised in Table 3.

### 3.4. Secondary Outcomes

No secondary caries was detected in any restoration. Postoperative hypersensitivity occurred in two participants (one in each group) and resolved spontaneously. No serious adverse events were observed. OHIP-14 scores showed high satisfaction with no inter-group differences. The mean total OHIP-14 score was 8.4 (±3.2) for the PICN group and 9.1 (±3.8) for the self-curing composite group (*p* = 0.42, paired *t*-test).

### 3.5. Summary of Findings

PICN hybrid ceramic restorations (VITA Enamic) demonstrated superior marginal integrity and lower dye penetration compared with self-curing composite (Stela) in both carious and abfraction cervical defects of professional ballet dancers over 24 months. Self-curing composite performed worse in abfraction defects (30% failure) than in carious defects (20% failure), whereas PICN showed 0% failure regardless of defect type.

Temporally, no PICN failures occurred at any time point. In the self-curing composite group, the first Charlie scores appeared at 12 months (10% in both defect types), progressing to Delta at 24 months (10% in carious, 10% in abfraction). Postoperative hypersensitivity occurred in two participants at 6 months and resolved spontaneously by 12 months.

## 4. Discussion

### 4.1. Principal Findings in Context

PICN restorations showed no clinical failures (USPHS Charlie/Delta) in either carious or abfraction defects throughout the observation period, with excellent marginal integrity and minimal dye penetration. In contrast, the bioactive self-curing composite exhibited cumulative failure rates of 20% in carious defects and 30% in abfraction defects at 24 months, with significantly higher dye penetration. Within the self-curing group, abfraction defects performed worse than carious defects, suggesting that non-carious cervical lesions pose a greater challenge for polymer-based direct restorations.

It is important to note that the two treatment arms differed not only in the restorative material but also in the clinical protocol (indirect, two-visit, laboratory-fabricated inlay bonded with dual-cure resin cement versus direct, single-visit, chairside self-curing composite). Therefore, the observed differences in performance should be interpreted as reflecting the overall restorative protocol rather than intrinsic material superiority alone.

It is important to reiterate that the comparisons between defect types within the self-curing group were not powered a priori and are exploratory in nature; therefore, they should be interpreted with caution.

### 4.2. Performance of PICN Hybrid Ceramic

The excellent performance of PICN (VITA Enamic) in the cervical region can be explained by its unique microstructure. PICN belongs to the class of polymer-infiltrated ceramic network materials, which consist of a porous feldspar ceramic network infiltrated with a polymer matrix [12]. This structure provides an elastic modulus (approximately 30 GPa) that closely mimics that of dentin and enamel, thereby reducing stress concentration at the tooth-restoration interface during cyclic loading, as demonstrated in vitro by Comba et al. [9]. In our study, the absence of any PICN restoration loss or Charlie-rated marginal defects at 24 months aligns with previous long-term clinical data. Oudkerk et al. [10] reported excellent 5-year wear resistance of PICN Computer-Aided Design and Computer-Aided Manufacturing (CAD-CAM) restorations in patients with severe tooth wear, and Kanaan et al. [13] found that indirect restorations, including PICN, outperformed direct composites in high-stress situations. A recent systematic review and meta-analysis by Alghauli et al. [11] confirmed low complication rates and high survival of resin-matrix ceramics, with an estimated annual failure rate of less than 2% for PICN-based restorations. Similarly, Banh et al. [14] reported favourable long-term longevity for PICN and zirconia-reinforced lithium silicate restorations. In the cervical region specifically, the indirect technique used for PICN restorations allows for precise marginal adaptation and the use of dual-cure resin cements, which provide stronger and more durable adhesion than direct composite placement [4,5]. Mahrous et al. [15] demonstrated that resin-matrix ceramics also exhibit good colour stability over time, an important aesthetic consideration for anterior cervical restorations. Bayraktar et al. [16] confirmed that PICN materials have superior wear characteristics and microhardness compared to many other CAD/CAM materials. Tissanavasoontra et al. [17] showed that PICN materials can be reliably repaired when necessary, a practical advantage in clinical practice. Finally, Bagratuni et al. [18] reported favourable 24-month clinical performance of PICN implant-supported crowns, further supporting the material’s reliability under occlusal loading. Taken together, these data provide strong evidence that PICN hybrid ceramics are well-suited for cervical restorations, particularly in patients with high occlusal loads.

### 4.3. Performance of the Bioactive Self Curing Composite (Stela)

Stela is a chemically cured bulk-fill composite that utilises a hydroperoxide-based initiator system, which virtually eliminates polymerisation shrinkage stress and achieves a gap-free interface in vitro [21]. Beyond its self-curing behaviour, Stela incorporates ion-releasing fillers (ionglass™) that provide bioactive properties. In vitro studies have demonstrated that such materials can release calcium, phosphate, and fluoride ions over time, and bioactive ion release has been associated with reduced bacterial activity and potential remineralisation [22]. Several clinical studies have evaluated Stela in posterior restorations. Loguercio et al. [23] reported an 18-month multicenter double-blind RCT showing that Stela performed comparably to a conventional light-cured composite in Class I and II cavities, with no significant differences in marginal adaptation or secondary caries. The same group [24] also published 6-month results confirming good clinical performance. Salem and Agila [25] found acceptable 6-month clinical outcomes for Stela in posterior teeth. However, these studies focused on occlusal restorations, where compressive stresses dominate. In our cervical restorations, especially in abfraction defects, the material is subjected to bending and tensile stresses—a more demanding environment. The higher failure rate of Stela in abfraction defects (30%) compared to carious defects (20%) likely reflects this difference. For comparison, Loguercio et al. reported no significant differences between Stela and conventional light-cured composites in Class I and II cavities at 18 months, suggesting that the cervical region—particularly abfraction lesions—imposes greater mechanical demands than occlusal restorations [23,24].

In vitro studies provide additional insights. Thadathil Varghese et al. [26] compared self-cure and dual-cure composites and found that while self-cure materials reduce polymerisation stress, their flexural strength and fracture toughness are slightly inferior to those of well-cured light-cure composites. Laporte et al. [27] evaluated the mechanical and physico-chemical properties of three polymer-based direct restorative materials and noted that self-cure composites may have lower antibacterial properties and reduced long-term stability under cyclic loading. Fibryanto et al. [28] reported that self-cure composites have higher volumetric polymerisation shrinkage compared to some light-cure alternatives, which could contribute to marginal gap formation over time. Albelasy et al. [23] examined internal adaptation and micromorphology of a new self-cure resin composite (Stela) and confirmed excellent initial adaptation, but long-term clinical data in high-stress regions are still lacking. Aliberti et al. [22] studied ionic release from restorative materials and noted that bioactive properties may be beneficial for caries prevention, but Stela does not claim the best bioactive ion release. Çarıkçıoğlu et al. [29] evaluated colour stability of self-cure bulk-fill composites and found acceptable results, but this does not address mechanical durability. Our clinical data suggest that the self-curing mechanism, which eliminates polymerisation shrinkage stress and creates a gap-free interface, may be insufficient when the restoration is repeatedly flexed at the cervical margin, particularly on sclerotic dentin typical of abfraction lesions. It is plausible that the suboptimal bond to hypermineralised dentin, combined with ongoing flexural stresses, may overwhelm the material’s resistance to marginal failure.

### 4.4. Specific Challenges of Abfraction Defects in Ballet Dancers

Abfraction defects are non-carious cervical lesions resulting from excessive occlusal forces that cause flexure and microfracture of enamel and dentin at the cemento-enamel junction [4,5]. Ballet dancers, due to constant tension of facial and masticatory muscles and parafunctional clenching, are particularly susceptible to these defects [2,3,8]. Our baseline data showed that the prevalence of abfraction lesions was high (19 out of 40 defects), and these lesions had a worse prognosis with the self-curing composite. The sclerotic, hypermineralised dentin surface of abfraction defects is thought to be challenging for reliable bonding [5,7]. The self-curing composite relies on its proprietary primer to initiate setting and adhesion; while this system works well on sound dentin, its performance on sclerotic dentin has not been extensively studied. In contrast, PICN restorations were luted with a dual-cure resin cement after acid etching of the cavity, providing a more predictable and durable bond even on sclerotic dentin. Furthermore, the absence of mechanical preparation for abfraction defects in the PICN group preserved the natural retentive geometry of the lesion, which may have contributed to the superior marginal seal observed. Clinicians should be aware that abfraction lesions in high-risk patients may require more robust restorative solutions than direct self-cure composites. Because carious and abfraction lesions differ in etiology, substrate characteristics, and biomechanical behavior, they were analyzed separately as pre-specified subgroups.

### 4.5. Clinical Implications for Ballet Dancers and Athletes

Our findings have direct clinical relevance for the management of cervical defects in professional dancers and, by extension, other patients with heavy occlusal loads (e.g., athletes, bruxers). For abfraction defects, the indirect PICN restoration protocol may be considered a favorable option based on its 24-month performance in this study. For carious cervical defects, the self-curing composite may be considered a less invasive, light-free alternative in patients who cannot tolerate lengthy light-curing procedures, but patients should be informed of a 20–30% risk of marginal deterioration over 24 months. Regardless of material choice, regular 6-month recalls are essential to detect early marginal gaps and prevent secondary caries. The use of protective occlusal splints, as advocated by Needleman [6], should be strongly recommended for ballet dancers to reduce parafunctional loads and prolong the lifespan of any restoration.

### 4.6. Strengths and Limitations

This study has several strengths. It is the first randomised controlled trial directly comparing a PICN hybrid ceramic with a modern self-curing composite in cervical defects. The split-mouth design eliminated inter-patient variability and allowed paired statistical analyses. The unique population of professional ballet dancers provides high external validity for other patient groups with heavy occlusal loads. Blinded outcome assessment, CONSORT-compliant reporting [14], and adherence to OHStat guidelines [15] enhanced internal validity. The dropout rate was 0%.

Limitations include the relatively small sample size (40 defects, 20 per material); subgroup analyses by defect type had limited statistical power. Furthermore, subgroup analyses by defect type were not pre-specified in the sample size calculation; consequently, the *p*-values reported for these comparisons (e.g., *p* = 0.031 within the self-curing group) are descriptive and exploratory only. The 24-month observation period is short for drawing conclusions about long-term survival; longer follow-up (3–5 years) is required to assess late failures, wear, and secondary caries. Operator blinding was not possible due to obvious differences in material handling (indirect vs. direct technique), although outcome assessor blinding was ensured. The exact mechanism of marginal deterioration (e.g., crack propagation vs. adhesive failure) was not directly visualised in this clinical study; we relied on indirect measures (dye penetration, marginal integrity). Our results may not generalise to other PICN or self-curing composites with different formulations. Nevertheless, because our study was conducted exclusively in professional ballet dancers, the findings should not be directly extrapolated to the general population without further research in other cohorts. Furthermore, the cavity preparation protocol differed between groups: abfraction defects received no mechanical preparation in the PICN group (to preserve the natural retentive shape), whereas the self-curing composite was placed after conventional adhesive preparation. This difference reflects the distinct clinical workflows for indirect versus direct restorations and could theoretically influence outcomes. However, for carious defects, where both materials were placed after comparable caries removal, PICN still demonstrated superior performance (0% vs. 20% failure), suggesting that the material itself is the dominant factor. Nevertheless, this difference in preparation remains a potential confounding factor and should be considered when interpreting the results. Moreover, we did not directly measure occlusal loading in the participants; the characterization of ballet dancers as a high-load population was based on literature and clinical observation rather than on individual bite force recordings. This should be considered when interpreting the external validity of the findings. Additionally, the two treatment arms differed in multiple aspects (indirect vs. direct workflow, number of visits, adhesive protocol, and marginal adaptation technique). Consequently, the observed differences cannot be attributed solely to the intrinsic properties of the restorative materials but rather to the complete restorative protocols. This should be considered when interpreting the comparative outcomes.

### 4.7. Future Research Directions

Given the single-center design and modest sample size, larger multi-center trials are needed before definitive clinical recommendations can be made. Several avenues for further research are warranted. Longer-term prospective studies (3–5 years) with larger sample sizes are needed to confirm the durability of PICN in cervical defects and to evaluate the fate of self-curing composite restorations beyond 24 months. Biomechanical finite element modelling could simulate the stress distribution at the cervical margin of PICN versus composite restorations under cyclic loading, explaining the mechanistic basis for our clinical observations. Studies focusing specifically on self-curing composites on sclerotic dentin, with improved adhesive protocols (e.g., selective enamel etching, use of universal adhesives), are needed to enhance bond strength in abfraction defects. Cost-effectiveness analyses comparing the higher initial cost of CAD-CAM PICN inlays versus the lower chair-time and material cost of direct self-curing composites would inform clinical decision-making. Finally, translation to other high-risk populations (e.g., patients with severe bruxism, cerebral palsy, or sports-related trauma) would validate the external applicability of our findings.

## 5. Conclusions

This 24-month split-mouth randomised controlled trial provides the first clinical This 24-month split-mouth RCT in professional ballet dancers provides the following key findings:

1. The indirect PICN restoration protocol (VITA Enamic) showed zero clinical failures (USPHS Charlie/Delta) and excellent marginal integrity in both carious and abfraction cervical defects over 24 months.
2. The direct self-curing composite protocol (Stela) had cumulative failure rates of 20% (carious) and 30% (abfraction), with significantly worse outcomes in abfraction lesions.
3. For professional ballet dancers (and, with caution, other patients with high occlusal loads), the indirect PICN protocol may be considered a preferable option for cervical restorations, especially for abfraction defects. The self-curing composite may be an acceptable, light-free alternative for carious defects when light curing is problematic, but patients should be informed of a substantial risk of marginal deterioration.
4. Longer-term studies (3–5 years) with larger sample sizes are required to confirm the durability of both protocols and to evaluate the clinical relevance of gap-free self-curing mechanisms under cyclic cervical loading. Extrapolation to the general population is not supported by this study.

## Figures and Tables

**Table 1:** Baseline characteristics (*n* = 20 patients).

Characteristic	Total (*n* = 20)
Age (years), mean ± standard deviation (SD)	34.2 ± 8.7
Female/Male	14/6
Professional experience (years)	18.4 ± 6.2
OHI S, median (Q1–Q3)	1.5 (1.2–2.0)
DMFT, median (Q1–Q3)	5.0 (3.0–8.0)
Defect type distribution: carious/abfraction (within the 40 defects)	21/19
Defect type per material	PICN—carious 11, abfraction 9; self curing—carious 10, abfraction 10

**Table 2:** Marginal integrity by defect type over 24 months (number of restorations, %).

Defect Type	Material	Time	Alpha	Bravo	Charlie	Delta	Failure Rate (%)	95% CI	*p* Value ^1^
Carious (*n* = 21)	PICN (*n* = 11)	6 m	11 (100)	0	0	0	0	0.0–28.5	—
		12 m	10 (91)	1 (9)	0	0	0	0.0–28.5	—
		24 m	10 (91)	1 (9)	0	0	0	0.0–28.5	0.083 ^2^
	Self-curing (*n* = 10)	6 m	9 (90)	1 (10)	0	0	0	0.0–30.1	—
		12 m	8 (80)	1 (10)	1 (10)	0	10	0.5–40.4	—
		24 m	7 (70)	1 (10)	1 (10)	1 (10)	20	5.7–51.0	—
Abfraction (*n* = 19)	PICN (*n* = 9)	6 m	9 (100)	0	0	0	0	0.0–33.6	—
		12 m	9 (100)	0	0	0	0	0.0–33.6	—
		24 m	8 (89)	1 (11)	0	0	0	0.0–33.6	0.052 ^2^
	Self-curing (*n* = 10)	6 m	9 (90)	1 (10)	0	0	0	0.0–30.1	—
		12 m	7 (70)	2 (20)	1 (10)	0	10	0.5–40.4	—
		24 m	5 (50)	2 (20)	2 (20)	1 (10)	30	11.9–54.3	0.031 ^3^

PICN = polymer-infiltrated ceramic network; CI = confidential interval. ^1^ *p*-values are adjusted for two co-primary outcomes using the Holm–Bonferroni method (α = 0.025). ^2^ Fisher’s exact test comparing PICN vs. self-curing for the same defect type at 24 months (PICN failure 0% vs. self-curing failure rate, *p* = 0.083 for carious, *p* = 0.052 for abfraction—not significant, but trend consistent). ^3^ Chi-square test comparing failure rates between carious and abfraction defects within self-curing group at 24 months. Exploratory comparison only; *p*-value is uncorrected for multiple testing. After applying the Holm–Bonferroni correction for the two co-primary outcomes (α = 0.025), this difference is not statistically significant.

**Table 3:** Dye penetration (positive) by defect type over 24 months.

Defect Type	Material	6 Months	12 Months	24 Months	*p* Value (24 Months) ^1^
Carious	PICN	0/11 (0%)	0/11 (0%)	1/11 (9%)	0.317
	Self curing	1/10 (10%)	2/10 (20%)	3/10 (30%)	
Abfraction	PICN	0/9 (0%)	1/9 (11%)	1/9 (11%)	0.048
	Self curing	1/10 (10%)	4/10 (40%)	6/10 (60%)	

^1^ McNemar’s test for paired comparison within defect type (PICN vs. self-curing at 24 months). Based on 9 evaluable pairs for abfraction defects. *p*-values are adjusted for two co-primary outcomes using the Holm–Bonferroni method (α = 0.025). The absolute difference in dye penetration at 24 months for abfraction defects was 49% (95% CI: 13–85%), favouring PICN.

## Data Availability

The datasets generated and analyzed during the current study are not publicly available due to patient privacy and institutional data protection policies. An anonymized minimal dataset is available from the corresponding author upon reasonable request and after approval by the Institutional Ethics Committee.

## Abbreviations

The following abbreviations are used in this manuscript:

RCT	Randomized Controlled Trial
PICN	Polymer-Infiltrated Ceramic Network
USPHS	United States Public Health Service
OHIP-14	Oral Health Impact Profile (14-item)
DMFT	Decayed, Missing, and Filled Teeth index
CONSORT	Consolidated Standards of Reporting Trials
OHStat	Oral Health Statistics guidelines
ITT	Intention-to-Treat
CI	Confidence Interval
SD	Standard Deviation
DEJ	Dentino-Enamel Junction
CAD/CAM	Computer-Aided Design/Computer-Aided Manufacturing
vs.	versus
